# The Placenta as a Source of Human Material for Neuronal Repair

**DOI:** 10.3390/biomedicines12071567

**Published:** 2024-07-15

**Authors:** Alessia Dallatana, Linda Cremonesi, Francesco Pezzini, Gianluca Fontana, Giulio Innamorati, Luca Giacomello

**Affiliations:** Department of Surgical Sciences, Dentistry, Gynecology and Pediatrics, University of Verona, 37134 Verona, Italy; alessia.dallatana@univr.it (A.D.); linda.cremonesi@univr.it (L.C.); francesco.pezzini@univr.it (F.P.); gianluca.fontana@univr.it (G.F.); luca.giacomello@univr.it (L.G.)

**Keywords:** placenta, mesenchymal stem cells, neuronal differentiation, extracellular vesicles, extracellular matrix, regenerative medicine

## Abstract

Stem cell therapy has the potential to meet unsolved problems in tissue repair and regeneration, particularly in the neural tissues. However, an optimal source has not yet been found. Growing evidence indicates that positive effects produced in vivo by mesenchymal stem cells (MSCs) can be due not only to their plasticity but also to secreted molecules including extracellular vesicles (EVs) and the extracellular matrix (ECM). Trophic effects produced by MSCs may reveal the key to developing effective tissue-repair strategies, including approaches based on brain implants or other implantable neural electrodes. In this sense, MSCs will become increasingly valuable and needed in the future. The placenta is a temporary organ devoted to protecting and supporting the fetus. At the same time, the placenta represents an abundant and extremely convenient source of MSCs. Nonetheless, placenta-derived MSCs (P-MSCs) remain understudied as compared to MSCs isolated from other sources. This review outlines the limited literature describing the neuroregenerative effects of P-MSC-derived biomaterials and advocates for exploiting the potential of this untapped source for human regenerative therapies.

## 1. Introduction

Neurogenesis is determined by sequences of extracellular stimuli. The commitment of neural stem cells (NSCs) occurs during the journey from neuronal crests to the formation of the complex architecture at the engraftment site. Progenitor NSCs can also be found in the adult brain although in very small amounts. Since harvesting NSCs from the adult brain is highly invasive [1,2], alternative strategies are needed to obtain the large amount of cells required for the treatment of human disorders affecting the neural system [3,4].

The human placenta is a potential source of precious biological material for regenerative purposes which could provide not only a very abundant source of mesenchymal stem cells (MSCs), but also of extracellular vesicles (EVs) and the extracellular matrix (ECM). In this review, we collected experimental evidence supporting the potential of placenta-derived MSCs (P-MSCs), placenta-derived EVs (P-EVs) and placenta-derived ECM (P-ECM) to fulfill the needs for regenerative approaches. Larger efforts have been dedicated to study MSCs from other sources. Adding to the limited studies discussed below, a more thorough investigation of placenta-derived biomaterials would certainly facilitate the design of practical and broadly available innovative approaches to neural tissue repair.

MSCs are adult stem cells characterized by the capacity to differentiate into multiple cellular lineages, the ability to self-renew, low telomerase activity, and immunomodulatory properties. MSCs in the human body are widely distributed, being present in the bone marrow, adipose tissue, and other tissues such as birth tissues [5]. 

Increasing knowledge about MSCs’ biological features is opening the way to novel therapeutic avenues for neural disorders. In the meantime, human pre-clinical studies based on MSC-cell therapy to treat neurological disorders, such as spinal cord injury (SCI), Multiple Sclerosis (MS), and others, have been carried out or are still ongoing [3,6,7]. Moreover, the increasing availability of breakthrough implantable neuronal interfaces and nanomaterials for neuronal repair has expanded the possible strategies to restore neurological functions, including combined approaches with cell-based therapies [8,9,10]. The most advanced brain chip technology is developing advanced electrodes to replace hard electrode mode and simulate the brain’s internal environment with hydrogels. Nonetheless, long-term inflammatory response and scar formation are difficult to avoid, and an efficient brain–computer interface would likely require combining with exogenous biological material [11]. It is easy to predict that the medical demand for ethically obtained and abundant sources of biomaterials will dramatically increase to allow the large-scale production of non-rejectable implants. P-MSCs could be the key to overcoming similar limitations.

**Figure 1:**
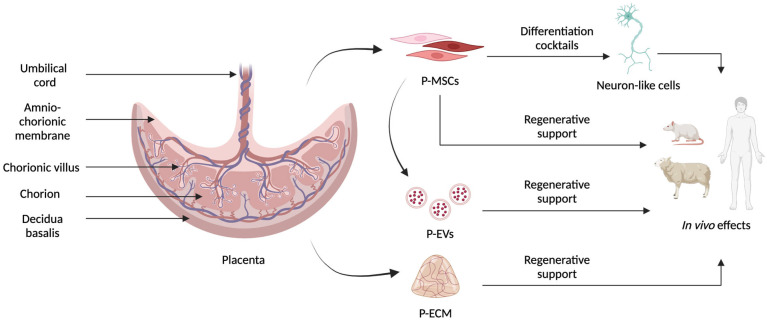
Schematic representation of the different tissues present in the placenta and of the different materials that can be obtained to develop innovative neuroregenerative therapies. P-MSCs in different colors represent the heterogenicity of cells that can be extracted from placental tissue.

The placenta provides the fetus with nourishment, oxygen, and protection from the mother’s immune response. It has both a maternal and a fetal origin and contains genetic material from both individuals [12]. Since it is usually discarded after birth, term stage placenta represents a rich source for MSCs that can be extracted from different parts of this organ, namely, the chorionic villi, decidua basalis, amniotic membrane, chorionic plate, and Wharton’s jelly, with the last three being the best in terms of proliferation [13], as schematized in Figure 1. P-MSCs can be collected without the invasive procedures or ethical implications associated with other sources. P-MSCs also have a higher proliferation rate, motility, survival, and expansion potential than other adult MSCs, like bone marrow-derived MSCs or adipose-derived MSCs [14]. 

Several protocols have been developed to extract P-MSCs, involving enzymatic digestion, explant culture, and perfusion. Enzymatic digestion exploits the action of dispase, dnase, collagenase, and trypsin which, respectively, digest P-ECM proteins, degrade free DNA preventing it from causing extracted cell clumping, digest collagen in P-ECM, and break cell–cell interactions allowing for the obtainment of single cell suspensions. Explant culture protocols involve a first brief digestion of the tissue which is then placed directly on culture supports like flasks or multiwell plates. The perfusion method is not recommended for the whole placenta as it is too time-consuming but is the most used method for umbilical cord MSC extraction from Wharton’s jelly. 

To confirm that cultured cells are P-MSCs, a characterization step requires osteogenic, chondrogenic, and adipogenic differentiation, immune-phenotyping with flow cytometry, and RT-PCR for MSC markers [14,15]. 

One limit of the literature we analyzed is that an exhaustive characterization of the cells isolated is not always present, particularly in older publications.

Although P-MSCs’ multipotency has been largely demonstrated [16], whether they should be considered pluripotent and can successfully be trans-differentiated to another embryonic lineage remains debated [17]. 

Over the last decades, the availability of increasingly sophisticated methodologies, and the awareness that morphology and marker expression are insufficient to document neural properties, have raised the level of experimental validations required to endorse a successful differentiation toward the neurogenic lineage [18]. Nonetheless, positive neurotrophic and neuroregenerative effects following in vivo administration of P-MSCs have been described in several animal models of human neurological disorders, even if the mechanism of action remains to be clarified, as discussed in the last paragraph. Following engraftment in the area of injury, P-MSCs have been reported to elicit trophic effects that may contribute to the endogenous repair process. Among the various hypothetical mechanisms mediated by P-MSCs, the secretion of immunomodulatory molecules is an important aspect that has not been considered in the present study and has been extensively reviewed elsewhere [19]. More recently, P-EVs and P-ECM attracted growing attention as putative mediators of neurotrophic processes and are reviewed in the following paragraphs. 

We conducted extensive online research by several search engines to collect insights about the applications of placenta-derived biomaterials in neuroregenerative therapies. This review summarizes the current experimental data available in this research field that appear relatively unexplored as compared to other sources of MSCs, i.e., bone marrow and adipose tissue. In this perspective, we discuss the advantages and disadvantages of this untapped source of biological material.

## 2. P-MSC-Derived Neuron-like Cells

Rather “basic” stimuli, such as cAMP-modulating agents and antioxidant substances (i.e., β-Mercaptoethanol or DMSO), administered to P-MSCs and other MSCs can produce dramatic transient morphological changes highly reminiscent of neuronal morphology [18]. Hou et al. observed a change in the shape of umbilical-cord-derived MSCs (UC-MSCs) after preinduction with β-mercaptoethanol and induction with butylated hydroxyanizole (BHA) and reported a concomitant expression of mature neuron markers, such as neuron-specific enolase (NSE), within 12 h [20]. Rooney et al. reported that an increase in intracellular cAMP in rat-derived bone-marrow MSCs (BM-MSCs) induced a transient neuronal morphology, associated with the increase in NSE and neurofilament (NF) in serum-free conditions 6 h after the stimulation. The absence of GAP43, a marker of neuronal outgrowth, led them to conclude that the protrusion in these cells did not correspond to the process characteristic of neuronal cells [21]. We reported a similar effect on chorion-derived MSCs that turned positive for immature neuronal markers, such as doublecortin, β tubulin III, and neuregulin [22]. Morphological changes related to toxic effects might have been interpreted as neurite formation in a former publication [18]. However, by documenting in time-lapse the reversion to a fibroblast-like shape the third day after cAMP induction, we proved that such neuron-like morphology can be transient and is not necessarily a prelude to cell death. We applied three different approaches to increase cAMP levels inside the cells, namely, forskolin, IBMX, and dbAMPc. All these methods led to the same effect with the same kinetics, peaking after 3 days from induction [22]. Despite the dramatic effect on cell morphology, it appears unrealistic that a rise in intracellular cAMP may by itself activate a complete differentiation program. 

During neural development, stem cells migrate to the correct location and assume the appropriate cellular morphology. Only a few cells remain confined in neurogenic niches and retain the basic properties of a stem cell. Ideally, differentiation protocols should surrogate the action of ancillary cells and the niche ECM creating an artificial neuroregeneration area. While more advanced approaches are being designed to reproduce the 3D neural stem cell niche [23] and could be applied to P-MSCs, so far, a variety of differentiation cocktails have been applied to commit MSCs toward functional neurons as reviewed by Jimenez-Acosta et al. [24]. Homemade or commercially available differentiation media are usually based on Alpha-MEM or DMEM enriched with supplements such as B27 and N2, growth factors such as EGF (Epidermal Growth Factor), FGF (Fibroblast Growth Factor), or BDNF (Brain-Derived Neurotrophic Factor), and chemical compounds such as retinoic acid, valproic acid, or forskolin [25,26,27,28,29].

To better mimic in vitro the physiological microenvironment, P-MSCs have been exposed to a conditioned medium derived from neuronal cells, such as the neuroblastoma SH-SY5Y cell line [30] or by exploiting direct co-culture with rat cerebellar astrocytes [31]. However, in both cases, only an increased expression of some neuronal genes was provided, without any functional validation. A higher degree of differentiation was reported on UC-MSCs with a neuronal-conditioned medium (NCM) obtained from primary cultured cells from the whole brain of a rat. This treatment induced the expression of NeuN, NF, and GFAP, and an inward current evoked by 1 mM glutamate was registered by electrophysiologic analysis [32]. By implementing the NCM with SHH (Sonic Hedgehog protein) and FGF, the same group reported the differentiation of dopaminergic neurons, based on the expression of tyrosine hydroxylase (TH), a dopaminergic neuron enzyme, and dopamine release in vitro. The same cells produced a partial improvement in motor coordination and balance when applied in vivo to the rat model of Parkinson’s Disease (PD) [33]. 

Another group reported that the exposure of Wharton’s jelly MSCs to human cerebrospinal fluid (CSF) induced the expression of neuronal (*Nestin* and *MAP2)* and glial (*GFAP*) genes, in a dose- and time-dependent manner, albeit no functional validation was reported [34]. Clearly, an intrinsic limit of these strategies based on CSF or neuronal preconditioned medium is the difficulty in obtaining a substantial amount for a virtually unlimited supply of MSCs. Furthermore, these approaches failed to provide convincing evidence of neuronal differentiation commitment according to present standards.

Other approaches attempted to improve neuronal differentiation by enhancing P-MSCs’ stemness or by expanding subpopulations of the precursors already present in primary cultures. In this context, the microenvironment plays a fundamental role and its effects are not limited to secreted factors. The interaction with other cells and the ECM, and the architecture and physicomechanical forces determined by these contacts, are all components determining cell fate [35]. Thanks to the fundamental self-assembling properties of cells, spheroids and organoids became extremely popular in the attempt to reproduce the niche and amplify progenitor cells. One example is described by Mukai et al. who derived neurospheres from UC-MSCs, reporting higher expression of neuronal, glial, and staminal markers in a heterogeneous pattern, as compared to the 2D culture [36]. Calzarossa et al. collected MSCs at the early stages of pregnancy (3–5 months) from chorionic villous sampling and amniocentesis. A preliminary morphological analysis of 2D primary culture performed immediately after isolation showed occasional branched cells resembling axons and dendrites already in adherent cells; however, this morphology was lost with passages. Following immunofluorescence, few cells were positive for ChAT and GABA, two specific neurotransmitter markers of functional neuronal fate commitment. A commercial neural stem cell expansion medium was applied to expand neuronal progenitors in neurosphere-like structures after 24 h of induction [37]. However, this 3D step could not expand NSCs since the aggregates did not proliferate. 

A more complex three-step protocol to commit mesenchymal cells to motor neurons was applied to P-MSCs obtained from amniochorionic membranes. Spheroids were successfully expanded in ultra-low attachment plates over the first two phases that were reported to produce first neural progenitors and then immature neurons. In the last step, cells were seeded on a polyornithine/laminin coat and stimulated with neurotrophic factors to achieve full differentiation into mature motor neurons. Maturation was documented by the expression of HB9 and VAChT, markers of motor neurons and cholinergic neurons, respectively, and by spontaneous electric activity, as demonstrated by the recording with a multi-electrode array plate assay [38]. 

Park and colleagues obtained spheroids starting from first-trimester chorionic villi-MSCs. An initial positivity to Nestin progressed to increased mature neural marker expression such as MAP2 and GFAP, up to 4 weeks of cultivation in vitro using a neural induction medium enriched with retinoic acid and N2. The efficacy of this approach was directly assessed in vivo. Following the Hypoxic–Ischemic insult (HI), Sprague–Dawley rats were transplanted with the differentiated cells achieving an improvement in locomotor activity. The presence of transplanted cells was confirmed by the localization of human nuclear antigen (HNa) in the transplantation site. Interestingly, evidence of colocalization with the neuronal dopaminergic marker TH might suggest differentiation of P-MSCs in the lesioned area [29]. The same group tested the effect of neural-progenitor-derived P-MSCs in a PD rat model causing the degeneration of dopaminergic neurons by 6-OHDA injection. Two weeks after the transplantation in the striatum of progenitor-derived MSCs, asymmetric rotational behavior was improved. Double immunoreactivity for TH and a human mitochondria marker in the graft site 12 weeks after the transplantation confirmed the in vivo differentiation of human-derived cells in the rat brain [39]. In the same experimental model, Kim et al. transplanted term-placenta MSCs induced to neural phenotype with FGF4 in spheroid cultures and drew the same conclusions. Following transplantation, affected rats improved their performance in a rotation test and showed partial recovery in dopaminergic function [40]. Taken together, these findings provide evidence of a possible direct neuronal-like differentiation of P-MSC in vivo. However, it has not been fully clarified whether the recovery of functional outcomes and the partial regeneration of lesioned tissue should be only ascribed to the neuronal-like P-MSCs or to the different trophic effects that such transplanted cells may exert locally. 

According to a search for relevant studies in PubMed, the interest in differentiating neural lineages from P-MSCs seems to have declined in the last decade. A search strategy that included the keywords “placenta”, “MSCs”, and “neuron differentiation” shows a peak of publication in 2010. The decrease in the following years may be related to the increased awareness that more compelling evidence beyond the morphological evaluation and the expression analysis of several markers is required to validate neuronal maturation and specific lineage differentiation. On the other hand, publications investigating the paracrine effects of these cells toward neuronal regeneration increased.

## 3. Placenta-Derived Extracellular Vesicles (P-EVs)

The physiological changes which the woman’s body undergoes during pregnancy are hypothesized to be mediated by soluble factors and EVs also released by the placenta (P-EVs). P-EVs are lipid bilayer structures ranging from 50 nm to several um which can be released by trophoblast cells, erythrocytes, and endothelial cells into the extracellular environment. They can contain RNA, DNA, and proteins that modulate the target cells’ bioactivity [41]. P-EVs may impact immune responses and the migration and invasion of placental cells during gestation as they are released into the maternal bloodstream in increasing levels as the pregnancy progresses [42]. In fact, pregnant women show an approximately 50-fold increase in their blood concentration [41]. In vivo studies suggested the beneficial paracrine effects of P-MSC transplantation in attenuating neuronal damage in animal disease models [43,44]. The nature of the responsible agents for neuroprotective effects has been investigated in a limited number of studies that highlighted the involvement of EVs in addition to soluble peptides [45,46]. The tail injection of MSCs derived from early gestation chorionic villi had a positive effect on functional recovery in animal models of spina bifida and multiple sclerosis. The effect could be ascribed to EVs that, isolated from the conditioned medium of the same cells, achieved comparable effects to whole cells, while EVs in depleted medium did not stimulate any response [44]. The intravitreal injection of amniotic membrane MSC-derived EVs, however, was reported to be beneficial in a rat model of glaucoma, showing a positive reduction in intraocular pressure compared to control groups. The effect was reproduced in vitro with an ARPE-19 cell line, suggesting that the exosomes acted directly on retinal pigment epithelial cells, promoting proliferation and providing protection from hypoxic insults [47].

Yang et al. analyzed exosomes derived from MSCs from different sources to treat SCI in animal models. The comparative study is based on the Basso–Beattie–Bresnahan (BBB) score [48] which considers different key points of animal movement such as joint movement, hindlimb and forelimb coordination, tail position, paw placement, stepping, and trunk stability. P-MSC-derived exosomes had the strongest effect on functional recovery. In addition, in vitro evidence showed P-MSC-derived EVs prevented the apoptosis of the human neuroblastoma cell line SH-SY5Y [49]. Hypoxia priming of P-MSCs has been proposed as a strategy to improve their regenerative capacity and their exosome performance [50]. De Laorden et al. utilized the amniotic-fluid-derived HMSC-AM cell line in co-culture with adult axotomized retinal ganglion cells (RGCs) [51]. Cell–cell interactions promoted axonal growth, an effect which was more pronounced when cells were incubated in hypoxic conditions. The functionality of the axon was proved not only by the expression of the synaptic vesicle glycoprotein 2A (SV2A) but also by the whole-cell patch clamp analysis of the sodium and potassium channels’ activity. However, the same study showed that by adding P-MSCs-derived conditioned media or separating cells with transwell supports, it was possible to increase axon length without significantly increasing the number of RGCs with an axon, meaning either direct cell contacts or higher concentrations of EVs are required. 

EVs collected at term deliveries from Wharton’s jelly MSCs exposed to hypoxia were also shown to prevent and resolve hypoxia-induced apoptosis on a mouse neuroblastoma cell line (N2a) [52] even though the underlying mechanisms were not clarified. A more accurate investigation of P-MSC-EVs’ action is offered by a study on a rat SCI model which reported increased neurological function scores administrating exosomes isolated from human P-MSCs [45]. The treatment increased the number of neurons, the levels of antioxidative factors (GSH, SOD, CAT), and the levels of the anti-inflammatory cytokine IL-10, while reducing the number of glial cells, the expression level of caspase 3, and the level of the oxidative factor MDA and the inflammatory cytokines IL-1b, IL18, and TNF-a. These findings suggest that P-MSC exosomes protect neural cells from oxidative stress and inflammation. A direct support to neuronal proliferation and the differentiation of progenitor cells was not demonstrated in this study, but in a very similar one showing that EVs promoted the activation and proliferation of endogenous neural progenitor cells, as reported by the increase in the number of cells positive for the specific markers SOX2, GFAP, PAX6, Nestin, SOX1, and Ki67 [43]. The analysis of the lesion area showed that the spinal cord ends of exosome-treated rats were fused to form a smooth nerve bundle structure while the rats in the control group had more fibrous and cystic tissues joining the same ends. Behavioral assessment of the rats’ hindlimb locomotor activity revealed that animals from exosome-treated and control groups had similar activities in the first 3 days after injury. Nevertheless, in the following 60 days, only the exosome-treated group kept improving while the control group remained at the initial damaged stage. Exosome-treated rats also recovered from the lesion-induced weight loss and showed an improvement in neurogenic bladder dysfunction. The same study also showed that P-MSC-EVs stimulated the proliferation of neural stem cells in vitro [43]. 

While the evidence of EVs’ efficacy in neurodegenerative diseases is increasing, there is still a relevant knowledge gap regarding whether EVs could be considered heterogeneous like the P-MSCs’ primary culture from which they are produced. Because this is a relatively novel field, most studies did not characterize the nature of EVs and poorly described how and where the tissue fragments were collected from the placenta. 

## 4. Placenta-Derived Extracellular Matrix (P-ECM)

The most recent technological advancements, such as 3D bioprinting, have expanded the use of biomaterial-based implants in the attempt to recreate in vitro the stem cell niche and thus more reliably study in 3D the MSCs’ behavior. Particularly, ECM components (such as integrins and collagen) and structural features such as matrix porosity impact cell migration and the differentiation of the cells within the scaffold, thus influencing stem cells’ regenerative potential [35]. ECM structural characteristics vary from each tissue of extraction and, in the case of placenta ECM, its applications have been reported as successful from wound healing to tissue regeneration [14]. Furthermore, P-MSCs, even if characterized by a greater proliferation compared to MSCs from other sources, are reported to not form teratomas [53]. In fact, the stem cells’ niche not only promotes cell proliferation but also controls it by avoiding uncontrolled growth [35].

The possibility of mimicking what happens in the niche in terms of spatiotemporal characteristics would allow for successfully translating P-ECM into effective stem-cell-based tissue engineering. 

Despite the fact that P-ECM immunomodulatory properties are well documented [54,55], its neuroregenerative or neuroprotective effects are largely overlooked. Placenta and neural tissues share key ECM components such as laminin and hyaluronic acid (HA) [56,57,58]. Laminin is a key protein within basement membranes [59] and has a specific role in neural tissue, influencing cell differentiation, migration, and attachment [59,60]. Laminin fosters neural circuit formation in neural tissues and aids nerve fiber regeneration [61]. In the placenta, laminin maintains the integrity of the trophoblast layer facilitating cell adhesion and migration [62,63]. Probably due to its abundance in this tissue, the placenta is one of the main sources of human laminin for biomedical applications [64]. HA is an abundant non-sulfated glycosaminoglycan endowed with anti-inflammatory properties [65,66]. In general, HA maintains tissue hydration and elasticity and provides mechanical support [67]. In neural systems, HA contributes to maintaining extracellular spaces and directs cell migration and signaling processes [68]. These characteristics suggested the exploitation of P-ECM in tissue engineering, including neuroregeneration. Seo et al. investigated the therapeutic potential of P-ECM on nerve damage using a sciatic nerve injury model [69]. The injection of P-ECM directly into the injury site promoted tissue regeneration by increasing the synthesis of GAP43 and Cdc2. GAP43 is a protein involved in axonal growth and guidance, and it is often used as a marker of neural plasticity being expressed in response to neural injury. On the other hand, Cdc2 controls various processes involved in mitosis such as chromosome condensation, the breakdown of the nuclear envelope, and spindle formation. Histological investigation confirmed that P-ECM promoted nerve fiber regeneration and myelination. Neurons treated with P-ECM had significantly longer neurite outgrowth, with thicker axonal myelin sheaths. A marked increase in GAP43 protein levels suggested that P-ECM may be directly involved in directing nerve regeneration [69].

Other studies highlighted the neuroprotective effects of P-ECM. For example, Farhadi et al. showed that P-ECM mitigates tinnitus symptoms induced by sodium salicylate exposure in rats [70]. Tinnitus is a disorder of the auditory system characterized by the perception of sound without any external stimuli. Phantom sounds like ringing, buzzing, or hissing are internally perceived without actual external sounds. The animals that received daily doses of P-ECM via intraperitoneal injections for up to 28 days showed improved Gap Pre-Pulse Inhibition of the Acoustic Startle (GPIAS) scores [70], which measure the reduction in a startle reflex by a gap of silence before a loud noise, indicating improved auditory processing. However, the study was limited to functional characterization, without histological or molecular investigations.

The neuroprotective effects of P-ECM were also demonstrated in a model of induced hypoxic–ischemic brain injury obtained by electrocauterizing the right common carotid [71]. Following the hypoxic–ischemic injury, rats lost brain regions involved in processing visual cues. Rats that received intraperitoneal injections of P-ECM before the induction of the injury showed shorter escape latencies during a maze test, highlighting higher cognitive function. The highest dose proved to be the most effective for maintaining cognitive skills like learning and memory. However, the administration of P-ECM after the injury did not achieve the same protective effect [71]. Although this study suggests that P-ECM injections could be used to prevent hypoxic–ischemic brain injury, the mechanisms of action are still elusive.

Mukherjee et al. [72] used placenta-derived laminin (extracted from a commercially available P-ECM, Placentrex) and tested its effects in vitro on neuronal differentiation using PC12 cells, a cell model for neuronal differentiation. The study showed that the interaction with laminin influenced PC12 cells transitions into neuron-like cells, enhancing neurite outgrowth. Measurements revealed longer neurites, with laminin distribution along the neuronal processes. Moreover, placental laminin enhanced the differentiation of PC12 cells while reducing proliferation, as demonstrated by the decrease in Ki67 expression. In the presence of an integrin receptor antagonist, the effect was lost implying a key role for the laminin–integrin interactions [72]. The scientific literature reports that the main integrins responsible for the interaction with laminin are α3β1, α6β1, and α7β1. The interaction of laminin–integrins can activate key signaling pathways, such as the FAK and PI3K/Akt pathways [73]. However, it is challenging to pinpoint specific biological effects because these signaling pathways are involved in many cellular responses to adhesion and environmental cues.

Because of the low immunogenicity of placenta extracts, several ongoing clinical studies are aimed to assess their regenerative capacity in human patients. P-ECMs like Laennec and Placentrex are already commercially available in some countries. When Laennec was administered via intravenous injections to human patients it was found to trigger the upregulation of hepcidin, a hormone that controls iron homeostasis, thus improving iron recycling in the liver [74]. Another study reported that Laennec administration led to a significant improvement in the general health status of elderly patients [75]. However, these studies only examined a limited number of patients and did not elucidate the pathways behind the effects observed. 

The broad application of P-ECM in regenerative medicine spans from wound-healing [76] to liver regeneration [74], and in vivo studies showed that P-ECM has neuroprotective effects [70,71]. An interesting study attempted to elucidate which components of P-ECM may have neuroregenerative properties by testing Laennec in a 5XFAD Alzheimer’s mouse model [77]. Mice exposed to the peptide fragment Aβ(25–35) for 3 days showed significant atrophy in the axons and dendrites of cortical neurons. Although intraperitoneal Laennec administration did not improve axonal density, the treatment on mice significantly improved object recognition memory and prevented the dendritic degeneration associated with Alzheimer’s disease. The authors found that components of P-ECM larger than 2 kDa were responsible for dendritic regeneration while the lower molecular weight fraction had no positive effects on dendrite regrowth [77]. The study claims that P-ECM administered intraperitoneally (Laennec) or orally (porcine placenta) had similar effects. The focus was primarily on observed outcomes rather than the mechanistic pathways and the study did not investigate how the biological signals of P-ECM could reach the mouse brain.

Jazayeri et al. investigated the effects of intraperitoneal injections of P-ECM in a mouse model of multiple sclerosis [78]. Administration of P-ECM for 31 days had a systemic effect which resulted in a significant decrease in serum concentration of the pro-inflammatory IL-23 and an increase in IL-27, which has a regulatory function that prevents unchecked inflammation [78], also reducing neural demyelination.

To date, the clinical adoption of P-ECM is limited to products designed for tissue repair and surgical procedures and those are only available in a few countries. To enable a broader adoption of P-ECM products, regulatory hurdles need to be overcome. For instance, MiMedx, the manufacturer of Axiofill^®^ (a P-ECM-based product), received a warning letter from the FDA for failing to meet minimal manipulation criteria. The FDA reported that Axiofill^®^ did not meet the regulatory classification requirements for human cell, tissue, or cellular- and tissue-based products (HCT/Ps) under section 361 of the Public Health Service Act. Although some P-ECM products obtained clearance as medical devices and obtained FDA approval through the 510(k) pre-notification process, these are not applied to neuroregenerative therapies. 

In essence, P-ECM has shown promising effects in helping to regenerate and protect neural cells. This highlights its potential in engineering neural tissues. While P-ECM’s ability to modulate the immune system is well known, its neuroregenerative capabilities have only recently been explored. Studies have demonstrated that P-ECM can enhance neural regeneration, mitigate hearing disorders, and improve cognitive functions in models of brain injury due to lack of oxygen and blood flow, as well as due to neurodegenerative diseases. Additionally, the low potential for immune rejection of P-ECM extracts and their regulatory approval in various countries underscore their potential for broader clinical applications. However, challenges remain in understanding the precise mechanisms of action and navigating regulatory requirements to ensure safety and effectiveness. 

## 5. Circumstantial Evidence of the Beneficial Effect Induced by In Vivo Administration of P-MSCs

Various groups reported the capability of human P-MSCs to restore neurological functions directly in vivo, without clarifying if the effect was the result of trans-differentiation, release of paracrine mediators, or secreted matrix. 

### 5.1. Spinal Cord Injury (SCI) Models

Applications of MSCs and P-MSC-EVs as a potential treatment for SCI have been extensively investigated and reviewed elsewhere [48,79]. Compared to studies on MSCs derived from other sources, few studies have been carried out by the transplantation of P-MSCs on SCI models. A functional recovery after injection of P-MSCs extracted from term placenta into the injured spinal cord was observed in Sprague–Dawley rat models of SCI. The recovery was determined by the analysis of somatosensory evoked potential and motor evoked potential in the rat hindlimbs, as well as by the evaluation of hindlimb functions with the BBB score [80]. However, the authors provided only a partial histopathological investigation and no information about the neuronal features of injected P-MSCs. 

A similar recovery of hindlimb functions was observed in a canine model of SCI following the transplantation of P-MSCs from term placenta that were cultured on linear-ordered collagen scaffolds (LOCS) to stimulate a better orientation and guidance of regenerating axons [81]. In transplanted dogs, locomotor hindlimb function was recovered better than in control animals up to 36 weeks post-injury. Neuropathological investigation documented that functional recovery was associated with increased axonal regeneration and remyelination in the lesion area. However, no P-MSCs could be detected in the injured tissues. 

More recently, Deng et al. injected the injured spinal cords of C57BL/6 mice with GFP-transfected P-MSCs, either grown in 2D or in spheroids [82]. As compared to 2D-P-MSCs- and PBS-treated mice, mice treated with 3D-P-MSCs showed that the 3D-P-MSCs ameliorated the tissue response, reducing the lesion cavity and astrogliosis at neuropathological investigation, and decreasing the dieback axonal distance (the axonal retraction from the lesion site) over time. The supporting survival of injured axons improved the functional outcome, in terms of locomotion and electrophysiological activity. Of note, in this study, P-MSC cells were detected in the lesion area up to 28 days after the transplantation. 

### 5.2. Congenital Defects of Spinal Cord: Myelomeningocele

Most literature reporting P-MSCs’ application for myelomeningocele (MMC) dealt with ovine models. Wang and colleagues utilized a combined therapeutical protocol in which a neuronal tube defect was induced in fetal lambs at 75 days of gestation and then repaired by intrauterine surgery at ~105 days [83]. The administration of 1 mL of collagen hydrogel containing 5 × 10^5^ P-MSCs extracted from the chorionic villus tissue of early gestational placenta (11–17 weeks) in the repaired lesion positively ameliorated motor functions. Specifically, four out six animals that received P-MSCs were able to ambulate independently, whereas all control animals remained immobilized. Motor function was quantified utilizing the Sheep Locomotor Rating (SLR) scale. Neuropathological investigation of the spinal cord 48 h after birth revealed an increased number of large neurons (LN) in lambs treated with P-MSCs. The number of neurons significantly correlated with the SLR score, suggesting a tight relationship. Since no P-MSC was detected, neither in the spinal cord nor in the surrounding tissue, the authors concluded that the improvement in the intrauterine surgery with P-MSC administration was possibly due to the beneficial, transient, paracrine effects of the cells. 

The P-MSCs’ therapeutic potential for MMC treatments could be further enhanced by culturing cells on clinical-grade porcine small intestinal submucosa (SIS)-derived ECM, creating a patch device to be applied on the site of lesion [84,85]. Again, a strong correlation between the LN and SLR score corroborated the hypothesis that the preservation of neuronal cells in the spinal cord may be the most relevant effect mediated by human P-MSCs [86]. Regardless of the mechanistic aspects responsible for the recovery, P-MSCs could improve the success of local surgical repair and avoid other potential detrimental effects secondary to the incomplete closure of the neuronal tube, such as amniotic fluid toxicity and hydrodynamic pressure. Notably, these beneficial effects were reproduced by preparing “clinical-grade” P-MSCs according to the Good Manufacturing Practice (GMP) standards required for the FDA approval of medical devices [87], thus indicating the suitability of this cell-based therapy for clinical trials. 

Comparable findings have been demonstrated in a Sprague–Dawley rat model of MMC, in which SCI was chemically induced by retinoic acid [88]. Also in this case, as compared to the ECM alone, the presence of P-MSCs seeded on SIS-ECM circular discs ameliorated the histopathological features of the injured area. The effect was quantified as a reduced compression of the spinal cord and a decreased density of apoptotic cells. However, as compared to studies focusing on MMC ovine models, no functional recovery tests were performed in vivo, making it difficult to evaluate a possible clinical implication. 

### 5.3. Optic Nerve Crush (ONC) Injury Models

Possible beneficial applications of P-MSCs in the recovery from the lesion of the optic nerve were investigated in ONC rat models. Injection into the left internal carotid artery of P-MSCs derived from the inner side of the chorioamniotic membrane of human term placenta partially restored both the mRNA and the protein expression levels of GAP43 in rat optic nerve extracts, one week and two weeks post-injury, respectively [89,90]. Moreover, one week after the cell administration, it was proved by RT-PCR and Western blotting that the expression of two human genes, namely *ERMN*, related to human retinal pigment epithelium, and *SRGAP2*, involved in neuronal migration and differentiation [89], was present in the treated eyes, indicating a possible engraftment of P-MSCs in the injured optic nerve. A partial recovery in the axon survival ratio was also observed in transplanted ONC rats as compared to non-injected/control ONC rats [90]. A parallel study on a model of R28 retinal ganglion cells (RGCs), which allows for investigating the axonal survival rate in vitro, indicated that the co-culturing of P-MSCs partially preserved RGCs from cell death following a hypoxic insult by CoCl_2_ treatment [90]. Further studies demonstrated that most of these beneficial effects of P-MSC administration in the ONC model might be mediated by BDNF and VEGF (Vascular Endothelial Growth Factor), and by the modulation of the NfkB pathway [91,92]. Despite the lack of visual recovery assays as well as a neuropathological investigation, these studies indicate that P-MSCs may support the local regeneration of the injured optic nerve traits, likely through neuroprotection and anti-inflammatory activities. 

### 5.4. Models of Neurodegenerative Disorder 

Different studies utilized multiple routes to administer P-MSCs derived from amniochorionic membrane on diverse rodent models of Alzheimer’s disease (AD) [93,94,95,96]. Improvements in memory and learning task performance were observed in all these studies. The cognitive amelioration was associated with a reduced Aβ plaque density in the hippocampus and different cortex area [94,95]. These findings seemed to correlate with changes in the recruitment of microglial cells and with a reduced inflammatory microenvironment. Yun and colleagues reported decreased expression of Aβ_1–42_, APP, and BACE as well as of microglial (Iba1), astrocytic (GFAP), and other inflammatory markers (iNOS and COX-2) [93]. Kim et al. observed the increased density of Iba+ microglia cells one week post-intravenous injection of P-MSCs in mice. After 12 weeks, the opposite trend was observed concurrently to an increased number of microglial cells expressing the phagocytic marker ED1, suggesting that P-MSCs’ transplantation modulates their phagocytic activity [96]. The precise role of P-MSCs in these disease models remains to be fully understood; since no transplanted cells were detected in the investigated brain areas, most of the beneficial effects are probably mediated by a paracrine mechanism [96]. 

The potential neuroregenerative capacity of P-MSCs was also investigated in a mouse model recapitulating Amyotrophic Lateral Sclerosis (ALS) [97]. Before administration, P-MSCs isolated from the amniotic membrane were cultured for 24 h in neurogenic induction medium (α-MEM containing 10% FBS, 0.1 µM dexamethasone, 0.5 µM linoleic acid, 10 ng/mL PDGF, and 10 ng/mL bFGF) and then in serum-free medium for a further 5 h. P-MSCs were administered systematically through the jugular vein using a multiple-administration protocol (three times, at 12, 14, and 16 weeks of age). The authors reported a significant extension of the lifespan and better motor performances of P-MSC-treated mice as compared to untreated mice. P-MSC transplantation significantly reduced astrogliosis, microgliosis, and the loss of motor neurons in the spinal cord ventral horns.

### 5.5. Global Cerebral Ischemia (GCI) 

In a rat model of Global Cerebral Ischemia (GCI) induced by a transient carotid artery occlusion, Kho and colleagues reported a phase of intrinsic neurogenesis, characterized by an increased number of doublecortin-positive cells in the hippocampus, one week after the injury. However, this phase was followed by an anergic state in which the neurogenesis no longer occurred [98]. Intravenous injection of P-MSCs one week after the injury seemed to sustain the intrinsic neurogenesis for the following week, delaying the anergic state. However, P-MSCs were scarcely detected in the hippocampus of transplanted animals, leaving the underlying mechanism unclear. In addition, since no functional recovery assessment was carried out, the clinical significance of such findings remained to be demonstrated. 

Taken together, these studies on animal models supported the beneficial effects of P-MSC transplantation. However, the engraftment and the survival of P-MSCs in the injured site as well as the evaluation of functional recovery are relevant issues which have been addressed only in a few studies (Table 1). Further attempts still deserve to be pursued to better understand the complex biological effects these cells may exert in vivo before their final clinical application in human trials.

## 6. Conclusions

A comparative study on P-MSCs of the first trimester and term placenta underlined how the commitment to different lineages depends on the gestational age and anatomical source of the placental tissue [13]. Most successful experimental approaches utilized earlier stages which are nonetheless definitely more difficult to obtain. The most promising approach for clinical applications remains fetal term P-MSCs that are reported to retain plasticity [99] and can be easily extracted from the umbilical cord and fetal membranes, i.e., amnion and chorion [100]. The potency towards the neural differentiation of cells obtained from these sources was preliminarily tested finding similar results [13]. P-MSCs collected both at the early stages of pregnancy (first trimester) [29,38] and at term were reported to include minoritarian populations of cells expressing neural-progenitor-related genes, such as Nestin and β tubulin III [31]. Even cells derived from a single placenta component are thus expected to offer distinct opportunities. A more accurate phenotyping could allow for discriminating within P-MSCs’ heterogeneity and improving their differentiation potential and the efficacy of their products. This will likely open up the possibility of widening the effective application of P-MSCs, P-EVs, and P-ECM to clinical practice, especially in the neuroregenerative field. Moreover, the possible synergistic effects of combining different placenta-derived biomaterials, such as P-ECM to encapsulate P-MSCs or P-EVs, should be investigated. 

The approach of new cutting-edge devices for neuronal tissue engineering will probably require innovative biomaterials to achieve a more efficacious engraftment with human tissue. In this view, the placenta represents an invaluable source of diverse biomaterials suitable for supporting neuronal recovery, as this review of scientific literature revealed.

In conclusion, the evidence reported in the literature as of today underlines the surmountable gap between placenta-derived materials and their application in neuroregenerative therapies.

## Figures and Tables

**Table 1:** Summary of the relevant studies about P-MSCs’ effects in animal models of neurodegenerative diseases.

Animal Models	Source of P-Biotools	P-MSC/EV Processing before Administration	Administration Route	Evaluation of Functional Recovery	Neuropathological Investigation	Engraftment/Differentiation Assessment	Ref.
***Spinal Cord Injury (SCI)***							
Sprague–Dawley rat	Decidual tissue of term placenta °	Differentiated (no details of medium provided)	In situ injection	- Somatosensory and motor evoked potentials in the rat hindlimbs - Evaluation of hindlimb functions (BBB scale)	H&E staining of injured spinal cords	Detection of P-MSC aggregates around the site of lesion	[80]
Beagle dog	Cotyledons of term placenta °	Seeded on LOCS	In situ injection	Improved locomotor hindlimb function recovery (Olby Scores)	Increased axonal regeneration and remyelination in the lesion area	N.A.	[81]
C57BL/6 mouse	Chorionic tissue from term placenta °	2D and spheroids culture	Stereotactically injected	Improved functional outcomes (electrophysiology; BMS locomotion; CatWalk step for coordination)	- Reduced lesion cavity and astrogliosis - Decreased axonal retraction from the lesion site	Detection of 3D P-MSCs in the lesion area up to 4 WPT	[82]
Sprague–Dawley rat	Term placenta °	Isolated exosomes	Intravenous injection	Increased neurological function scores (Motor Deficit Index test: hind-paw placing and stepping reflex evaluations)	Increased number of neurons, levels of antioxidative factors, and levels of anti-inflammatory cytokine IL-10 Reduced number of glial cells, expression of caspase 3, level of oxidative factor MDA, and levels of inflammatory cytokines	N.A.	[45]
Sprague–Dawley rat	Term placenta °	Isolated exosomes	Intravenous injection	Increased hindlimb locomotor activity; recovery from the lesion-induced weight loss; improved neurogenic bladder dysfunction	Increased number of cells positive for SOX2, GFAP, PAX6, Nestin, SOX1, and Ki67 in the spinal cord	N.A.	[43]
***Multiple Sclerosis (MS)***							
C57BL/6J Mouse	Chorioamniotic membrane of pre-term placenta °	Untreated cells + isolated extracellular vesicles	Intravenous injection	Improved myelin regeneration and reduced DNA damage in oligodendroglia population in the spinal cord	- Reduced oligodendroglia degeneration (decreased expression of TUNEL and SOX10 double positive cells) in spinal cord tissue sections - Significant reduction in myelin loss in the spinal cord (Luxol Fast Blue staining)	N.A.	[44]
***Optic Nerve Crush (ONC) injury***							
Sprague–Dawley rat	Chorioamniotic membrane of term placenta °; umbilical cord blood (UCBMSC)	Differentiated (aMEM + 10% FBS + hFGF4 + Heparin)	Injection (carotid artery)	N.A.	Slight effects in preserving the axon survival of UCBMSC	Evidence of human *ERMN* and *SRGAP2* expression	[89]
Sprague–Dawley rat	Chorioamniotic membrane of term placenta °	Differentiated (DMEM/F12 + 10% FBS + hFGF4 + Heparin)	Injection (carotid artery)	N.A.	N.A.	N.A.	[90]
Sprague–Dawley rat	Chorioamniotic membrane of term placenta °	Differentiated (DMEM/F12 + 10% FBS + hFGF4 + Heparin) and hypoxic insult	Subtenon injection	N.A.	Increased gene expression of neuronal (GAP43) and glial (GFAP) markers in nerve extracts	Detection of P-MSCs in the optic nerve tissue 4 WPT	[91]
Sprague–Dawley rat	Chorioamniotic membrane of term placenta °	Differentiated (DMEM/F12 + 10% FBS + hFGF4 + Heparin)	Subtenon injection	N.A.	Increased GAP43 expression and reduced Iba1 expression in the optic nerve	N.A.	[92]
***Myelomeningocele (MMC)***							
Sheep	Chorionic villus tissue of pre-term placenta °	Seeded on collagen hydrogel	In situ application	Improved motor function (SLR scale); 4 out 6 animals which received the PDMSC were able to ambulate independently	Increased number of large neurons in spinal cord of treated lambs	No long-lasting engraftment of P-MSCs	[83]
Sprague–Dawley rat	Chorionic villus tissue of pre-term placenta °	Seeded on SIS-ECM circular discs	In situ application	N.A.	- Reduced compression of the spinal cord - Decreased density of apoptotic cells	N.A.	[65]
Sheep	Chorionic villus tissue of pre-term placenta °	Seeded on SIS-ECM circular discs §	In situ application	Improved motor function (SLR scale)	- Increased number of large neurons - Increased gray matter and spinal cord areas	N.A.	[84,85,86,87]
***Ischemic Encephalopathy***							
Sprague–Dawley rat	Chorionic villus tissue of pre-term placenta °^,^*	- Spheroids culture - Differentiated (DMEM/F12, RA, N2) - Neuroblastoma-CM	Stereotactically injected (bilateral striata)	Improved motor and coordination abilities (Rotarod test)	Increased expression of neuronal dopaminergic (TH) and astrocytic (GFAP) markers in the sites of transplantation	Detection of P-MSCs in the lesion area up to 8 WPT	[29]
Sprague–Dawley rat	Amniotic membrane of term placenta °	Differentiated (aMEM + 10% FBS + hFGF4 + Heparin)	Intravenous injection	N.A.	Prolonged intrinsic neurogenesis (DCX+/BrdU+/Ki67+) and delayed anergic state	No direct evidence of differentiation in the tissue	[98]
***Parkinson Disease (PD)***							
Sprague–Dawley rat	Wharton’s jelly from umbilical cord	Differentiated (DMEM + 10%FBS + NCM + Shh + FGF8)	Stereotactically injected (striatum)	TH immunocytochemistry in grafted striatum	Increased TH immunoreactivity in the lesioned side of striatum	- Detection of TH positive cells in the implantation area - Colocalization of TH positive cells and human-specific nuclear antigen	[33]
Sprague–Dawley rat	Chorionic villus tissue of pre-term placenta °^,^*	- Spheroids culture - Differentiated (DMEM/F12, RA, N2) - Neuroblastoma-CM	Stereotactically injected (striatum)	- Amelioration of asymmetric rotational behavior - Partial recovery in dopaminergic function (^18^F-FP-CIT PET analysis)	Widespread TH expression in the injured striatum	Detection of human markers (hNA and hMT) and colocalization with neuronal markers (NeuN and TH) at 12 WPT	[39]
Sprague–Dawley rat	Amniotic membrane of term placenta °	Spheroids culture + hFGF4	Stereotactically injected	- Amelioration of motor deficits (rotation test; Rotarod; cylinder test) - Partial recovery in dopaminergic function (^18^F-FP-CIT PET analysis)	Increased TH immunoreactivity in the lesioned side of striatum	- Detection of human marker (HN) and colocalization with neuronal markers (TH, GABA, glutamate) up to 12 WPT	[40]
***Alzheimer Disease (AD)***							
Tg2576 transgenic (APPswe) Mouse	Amniotic membrane of term placenta °	Amniotic epithelial cell media + 10% FBS	Stereotactically injected	Improved learning and memory capacities (Morris WMT; Y-maze test)	- Amyloid plaques in the brains of Tg2576 mice - Reduced β-secretase activity	N.A.	[94]
C57BL/6J-APP mouse	Amniotic membrane of term placenta °	DMEM + 10% FBS	Intravenous injection	Attenuation of spatial learning and memory function deficits (WMT)	- Reduction in Aβ deposition - Reduction in oxidative stress	N.A.	[95]
Tg2576 transgenic (APPswe) Mouse	Amniotic membrane of term placenta °	Differentiated (aMEM + 10% FBS + hFGF4 + Heparin)	Intravenous injection	Improved memory performance from the 3rd to 4th trials (WMT)	- Reduced number of Aβ plaques - Increased number of activated microglia cells with phagocytic activity (co-expression of Iba1 and ED1 markers)	No detection of human cells 1 week post-injection in the treated area	[96]
ICR Mouse	Chorioamniotic membrane of term placenta °	Differentiated (aMEM + 10% FBS + hFGF4 + Heparin)	Intravenous injection	Improved learning and memory capacities (water maze test; probe test)	- Reduced anti-amyloidogenic effects (decreased expression of Ab1–42, APP, BACE) in hippocampus - Decreased expression of microglial (Iba1), astrocytic (GFAP) and inflammatory markers (iNOS and COX-2) in hippocampus	No direct evidence of differentiation in the tissue	[93]
***Amyotrophic lateral sclerosis (ALS)***							
hSOD1^G93A^ transgenic mouse	Amniotic membrane of term placenta	α-MEM + 10% FBS + dexamethasone + linoleic acid + PDGF + bFGF for 24 h and then processed without serum for 5 h	Intravenous injection (multiple administrations)	- Significant extension of lifespan - Better motor performances (Rotarod, PaGE test, CatWalk gait analysis)	- Preservation of motor neurons in the spinal cord ventral horns - Reduced microgliosis and astrogliosis compared to PBS-treated mice	- Some cell clusters detected in the spinal cord - No differentiation of transplanted cells into neurons or glia	[97]

° Term placenta, >37 weeks; pre-term placenta, 11–17 weeks; * chorionic villi of fetus failed to maintain heart beatings at 5–7 weeks after embryo transfer; § Clinical-grade culture according to the GMP standards; LOCS, linear-ordered collagen scaffolds; SIS-ECM, porcine small intestinal submucosa (SIS)-derived extracellular matrix (ECM); CM, conditional medium; N.A., not available; WPT, week post-transplantation; BBB, Basso–Beattie–Bresnahan locomotor rating scale; SLR, Sheep Locomotor Rating; WMT, water maze test; HN, human nuclear marker, hMt, human mitochondrial marker; GAP43, NeuN, DCX, neuronal markers; BrdU, Ki67, proliferation markers; GFAP, astrocytic marker; TH, dopaminergic marker of dopaminergic neurons; Iba1, microglial marker; iNOS, COX-2, inflammatory markers; NCM, neuronal-conditioned medium; Shh, Sonic hedgehog.

## Data Availability

Not applicable.

## References

[1] Ma D.K., Bonaguidi M.A., Ming G.L., Song H. (2009). Adult Neural Stem Cells in the Mammalian Central Nervous System. Cell Res..

[2] Matsubara S., Matsuda T., Nakashima K. (2021). Regulation of Adult Mammalian Neural Stem Cells and Neurogenesis by Cell Extrinsic and Intrinsic Factors. Cells.

[3] Genchi A., Brambilla E., Sangalli F., Radaelli M., Bacigaluppi M., Furlan R., Andolfo A., Drago D., Magagnotti C., Scotti G.M. (2023). Neural Stem Cell Transplantation in Patients with Progressive Multiple Sclerosis: An Open-Label, Phase 1 Study. Nat. Med..

[4] Kaminska A., Radoszkiewicz K., Rybkowska P., Wedzinska A., Sarnowska A. (2022). Interaction of Neural Stem Cells (NSCs) and Mesenchymal Stem Cells (MSCs) as a Promising Approach in Brain Study and Nerve Regeneration. Cells.

[5] Pittenger M.F., Discher D.E., Péault B.M., Phinney D.G., Hare J.M., Caplan A.I. (2019). Mesenchymal Stem Cell Perspective: Cell Biology to Clinical Progress. NPJ Regen. Med..

[6] Gavasso S., Kråkenes T., Olsen H., Evjenth E.C., Ytterdal M., Haugsøen J.B., Kvistad C.E. (2024). The Therapeutic Mechanisms of Mesenchymal Stem Cells in MS—A Review Focusing on Neuroprotective Properties. Int. J. Mol. Sci..

[7] Van den Bos J., El Ouaamari Y., Wouters K., Cools N., Wens I. (2022). Are Cell-Based Therapies Safe and Effective in the Treatment of Neurodegenerative Diseases? A Systematic Review with Meta-Analysis. Biomolecules.

[8] Doblado L.R., Martínez-Ramos C., Pradas M.M. (2021). Biomaterials for Neural Tissue Engineering. Front. Nanotechnol..

[9] Sun P., Li C., Yang C., Sun M., Hou H., Guan Y., Chen J., Liu S., Chen K., Ma Y. (2024). A Biodegradable and Flexible Neural Interface for Transdermal Optoelectronic Modulation and Regeneration of Peripheral Nerves. Nat. Commun..

[10] Yari-Ilkhchi A., Mahkam M., Ebrahimi-Kalan A., Zangbar H.S. (2024). Design and Synthesis of Nano-Biomaterials Based on Graphene and Local Delivery of Cerebrolysin into the Injured Spinal Cord of Mice, Promising Neural Restoration. Nanoscale Adv..

[11] Zhang J., Wang T., Zhang Y., Lu P., Shi N., Zhu W., Cai C., He N. (2022). Soft Integration of a Neural Cells Network and Bionic Interfaces. Front. Bioeng. Biotechnol..

[12] Burton G.J., Fowden A.L. (2015). The Placenta: A Multifaceted, Transient Organ. Philos. Trans. R. Soc. B Biol. Sci..

[13] Portmann-Lanz C.B., Schoeberlein A., Huber A., Sager R., Malek A., Holzgreve W., Surbek D.V. (2006). Placental Mesenchymal Stem Cells as Potential Autologous Graft for Pre- and Perinatal Neuroregeneration. Am. J. Obs. Gynecol..

[14] Biswas A., Rajasekaran R., Saha B., Dixit K., Vaidya P.V., Ojha A.K., Dhara S. (2023). Human Placenta/Umbilical Cord Derivatives in Regenerative Medicine—Prospects and Challenges. Biomater. Sci..

[15] Innamorati G., Fontana E., Steccanella F., Gandhi K., Bassi G., Zandonà V., Giacomello L. (2017). Pleiotropic Effects of Sphingosine-1-Phosphate Signaling to Control Human Chorionic Mesenchymal Stem Cell Physiology. Cell Death Dis..

[16] Li C.D., Zhang W.Y., Li H.L., Jiang X.X., Zhang Y., Tang P., Mao N. (2005). Mesenchymal Stem Cells Derived from Human Placenta Suppress Allogeneic Umbilical Cord Blood Lymphocyte Proliferation. Cell Res..

[17] Bhartiya D. (2013). Are Mesenchymal Cells Indeed Pluripotent Stem Cells or Just Stromal Cells? OCT-4 and VSELs Biology Has Led to Better Understanding. Stem Cells Int..

[18] Barnabé G.F., Schwindt T.T., Calcagnotto M.E., Motta F.L., Martinez G., de Oliveira A.C., Keim L.M.N., D’Almeida V., Mendez-Otero R., Mello L.E. (2009). Chemically-Induced RAT Mesenchymal Stem Cells Adopt Molecular Properties of Neuronal-Like Cells but Do Not Have Basic Neuronal Functional Properties. PLoS ONE.

[19] Papait A., Silini A.R., Gazouli M., Malvicini R., Muraca M., O’Driscoll L., Pacienza N., Toh W.S., Yannarelli G., Ponsaerts P. (2022). Perinatal Derivatives: How to Best Validate Their Immunomodulatory Functions. Front. Bioeng. Biotechnol..

[20] Hou L., Cao H., Wang D., Wei G., Bai C., Zhang Y., Pei X. (2003). Induction of Umbilical Cord Blood Mesenchymal Stem Cells into Neuron-Like Cells In Vitro. Int. J. Hematol..

[21] Rooney G.E., Howard L., O’Brien T., Windebank A.J., Barry F.P. (2009). Elevation of CAMP in Mesenchymal Stem Cells Transiently Upregulates Neural Markers Rather than Inducing Neural Differentiation. Stem Cells Dev..

[22] Innamorati G., Ridolfi G., Steccanella F., Bormetti A., Dallatana A., Bozzetto C., Ottoboni L., Di Chio M., Giacomello L. (2022). CAMP Response Element-Binding Protein Controls the Appearance of Neuron-Like Traits in Chorion Mesenchymal Cells. Front. Biosci.—Landmark.

[23] Ioannidis K., Angelopoulos I., Gakis G., Karantzelis N., Spyroulias G.A., Lygerou Z., Taraviras S. (2021). 3D Reconstitution of the Neural Stem Cell Niche: Connecting the Dots. Front. Bioeng. Biotechnol..

[24] Jiménez-Acosta M.A., Hernández L.J.R., Cristerna M.L.P., Tapia-Ramírez J., Meraz-Ríos M.A., Jiménez-Acosta M.A., Hernández L.J.R., Cristerna M.L.P., Tapia-Ramírez J., Meraz-Ríos M.A. (2022). Review: Neuronal Differentiation Protocols of Mesenchymal Stem Cells. Adv. Biosci. Biotechnol..

[25] Chen L., He D.M., Zhang Y. (2009). The Differentiation of Human Placenta-Derived Mesenchymal Stem Cells into Dopaminergic Cells in Vitro. Cell Mol. Biol. Lett..

[26] Talwadekar M., Fernandes S., Kale V., Limaye L. (2017). Valproic Acid Enhances the Neural Differentiation of Human Placenta Derived-Mesenchymal Stem Cells in Vitro. J. Tissue Eng. Regen. Med..

[27] Portmann-Lanz B., Schoeberlein A., Portmann R., Mohr S., Rollini P., Sager R., Surbek D.V. (2010). Turning Placenta into Brain: Placental Mesenchymal Stem Cells Differentiate into Neurons and Oligodendrocytes. Am. J. Obstet. Gynecol..

[28] Cortés-Medina L.V., Pasantes-Morales H., Aguilera-Castrejon A., Picones A., Lara-Figueroa C.O., Luis E., Montesinos J.J., Cortés-Morales V.A., De la Rosa Ruiz M.P., Hernández-Estévez E. (2019). Neuronal Transdifferentiation Potential of Human Mesenchymal Stem Cells from Neonatal and Adult Sources by a Small Molecule Cocktail. Stem Cells Int..

[29] Park S., Koh S.E., Maeng S., Lee W.D., Lim J., Lee Y.J. (2011). Neural Progenitors Generated from the Mesenchymal Stem Cells of First-Trimester Human Placenta Matured in the Hypoxic-Ischemic Rat Brain and Mediated Restoration of Locomotor Activity. Placenta.

[30] Zammit V., Brincat M.R., Cassar V., Muscat-Baron Y., Ayers D., Baron B. (2018). MiRNA Influences in Mesenchymal Stem Cell Commitment to Neuroblast Lineage Development. Noncoding RNA Res..

[31] Martini M.M., Jeremias T.D.S., Kohler M.C., Marostica L.L., Trentin A.G., Alvarez-Silva M. (2013). Human Placenta-Derived Mesenchymal Stem Cells Acquire Neural Phenotype Under the Appropriate Niche Conditions. DNA Cell Biol..

[32] Fu Y.S., Shih Y.T., Cheng Y.C., Min M.Y. (2004). Transformation of Human Umbilical Mesenchymal Cells into Neurons in Vitro. J. Biomed. Sci..

[33] Fu Y.-S., Cheng Y.-C., Lin M.-Y.A., Cheng H., Chu P.-M., Chou S.-C., Shih Y.-H., Ko M.-H., Sung M.-S. (2006). Conversion of Human Umbilical Cord Mesenchymal Stem Cells in Wharton’s Jelly to Dopaminergic Neurons In Vitro: Potential Therapeutic Application for Parkinsonism. Stem Cells.

[34] Farivar S., Mohamadzade Z., Shiari R., Fahimzad A. (2015). Neural Differentiation of Human Umbilical Cord Mesenchymal Stem Cells by Cerebrospinal Fluid. Iran. J. Child. Neurol..

[35] Augustine R., Dan P., Hasan A., Khalaf I.M., Prasad P., Ghosal K., Gentile C., McClements L., Maureira P. (2021). Stem Cell-Based Approaches in Cardiac Tissue Engineering: Controlling the Microenvironment for Autologous Cells. Biomed. Pharmacother..

[36] Mukai T., Nagamura-Inoue T., Shimazu T., Mori Y., Takahashi A., Tsunoda H., Yamaguchi S., Tojo A. (2015). Neurosphere Formation Enhances the Neurogenic Differentiation Potential and Migratory Ability of Umbilical Cord-Mesenchymal Stromal Cells. Cytotherapy.

[37] Calzarossa C., Bossolasco P., Besana A., Manca M.P., De Grada L., De Coppi P., Giardino D., Silani V., Cova L. (2013). Neurorescue Effects and Stem Properties of Chorionic Villi and Amniotic Progenitor Cells. Neuroscience.

[38] Gaggi G., Di Credico A., Guarnieri S., Mariggiò M.A., Ballerini P., Di Baldassarre A., Ghinassi B. (2022). Human Fetal Membrane-Mesenchymal Stromal Cells Generate Functional Spinal Motor Neurons in Vitro. iScience.

[39] Park S., Kim E., Koh S.E., Maeng S., Lee W.D., Lim J., Shim I., Lee Y.J. (2012). Dopaminergic Differentiation of Neural Progenitors Derived from Placental Mesenchymal Stem Cells in the Brains of Parkinson’s Disease Model Rats and Alleviation of Asymmetric Rotational Behavior. Brain Res..

[40] Kim H.W., Lee H.-S., Kang J.M., Bae S.-H., Kim C., Lee S.-H., Schwarz J., Kim G.J., Kim J.-S., Cha D.H. (2018). Dual Effects of Human Placenta-Derived Neural Cells on Neuroprotection and the Inhibition of Neuroinflammation in a Rodent Model of Parkinson’s Disease. Cell Transpl..

[41] Salomon C., Torres M.J., Kobayashi M., Scholz-Romero K., Sobrevia L., Dobierzewska A., Illanes S.E., Mitchell M.D., Rice G.E. (2014). A Gestational Profile of Placental Exosomes in Maternal Plasma and Their Effects on Endothelial Cell Migration. PLoS ONE.

[42] Nakahara A., Nair S., Ormazabal V., Elfeky O., Garvey C.E., Longo S., Salomon C. (2020). Circulating Placental Extracellular Vesicles and Their Potential Roles During Pregnancy. Ochsner J..

[43] Zhou W., Silva M., Feng C., Zhao S., Liu L., Li S., Zhong J., Zheng W. (2021). Exosomes Derived from Human Placental Mesenchymal Stem Cells Enhanced the Recovery of Spinal Cord Injury by Activating Endogenous Neurogenesis. Stem Cell Res. Ther..

[44] Clark K., Zhang S., Barthe S., Kumar P., Pivetti C., Kreutzberg N., Reed C., Wang Y., Paxton Z., Farmer D. (2019). Placental Mesenchymal Stem Cell-Derived Extracellular Vesicles Promote Myelin Regeneration in an Animal Model of Multiple Sclerosis. Cells.

[45] Jafari A., Khalatbary A.R., Taghiloo S., Mirzaie M.S., Nazar E., Poorhassan M., Akbari E., Asadzadeh M., Raoofi A., Nasiry D. (2023). Exosomes Derived from Human Placental Mesenchymal Stem Cells in Combination with Hyperbaric Oxygen Synergically Alleviates Spinal Cord Ischemia-Reperfusion Injury. Regen. Ther..

[46] Soleimani A., Oraee Yazdani S., Pedram M., Saadinam F., Rasaee M.J., Soleimani M. (2024). Intrathecal Injection of Human Placental Mesenchymal Stem Cells Derived Exosomes Significantly Improves Functional Recovery in Spinal Cord Injured Rats. Mol. Biol. Rep..

[47] Seong H.-R., Noh C.H., Park S., Cho S., Hong S.-J., Lee A.-Y., Geum D., Hong S.-C., Park D., Kim T.M. (2023). Intraocular Pressure-Lowering and Retina-Protective Effects of Exosome-Rich Conditioned Media from Human Amniotic Membrane Stem Cells in a Rat Model of Glaucoma. Int. J. Mol. Sci..

[48] Yang Z., Liang Z., Rao J., Lin F., Lin Y., Xu X., Wang C., Chen C. (2023). Mesenchymal Stem Cell-Derived Extracellular Vesicles Therapy in Traumatic Central Nervous System Diseases: A Systematic Review and Meta-Analysis. Neural Regen. Res..

[49] Kumar P., Becker J.C., Gao K., Carney R.P., Lankford L., Keller B.A., Herout K., Lam K.S., Farmer D.L., Wang A. (2019). Neuroprotective Effect of Placenta-Derived Mesenchymal Stromal Cells: Role of Exosomes. FASEB J..

[50] Pulido-Escribano V., Torrecillas-Baena B., Camacho-Cardenosa M., Dorado G., Gálvez-Moreno M.Á., Casado-Díaz A. (2022). Role of Hypoxia Preconditioning in Therapeutic Potential of Mesenchymal Stem-Cell-Derived Extracellular Vesicles. World J. Stem Cells.

[51] de Laorden E.H., Simón D., Milla S., Portela-Lomba M., Mellén M., Sierra J., de la Villa P., Moreno-Flores M.T., Iglesias M. (2023). Human Placenta-Derived Mesenchymal Stem Cells Stimulate Neuronal Regeneration by Promoting Axon Growth and Restoring Neuronal Activity. Front. Cell Dev. Biol..

[52] Joerger-Messerli M.S., Oppliger B., Spinelli M., Thomi G., di Salvo I., Schneider P., Schoeberlein A. (2018). Extracellular Vesicles Derived from Wharton’s Jelly Mesenchymal Stem Cells Prevent and Resolve Programmed Cell Death Mediated by Perinatal Hypoxia-Ischemia in Neuronal Cells. Cell Transpl..

[53] Pipino C., Shangaris P., Resca E., Zia S., Deprest J., Sebire N.J., David A.L., Guillot P.V., De Coppi P. (2013). Placenta as a Reservoir of Stem Cells: An Underutilized Resource?. Br. Med. Bull..

[54] Wang C., Li G., Cui K., Chai Z., Huang Z., Liu Y., Chen S., Huang H., Zhang K., Han Z. (2021). Sulfated Glycosaminoglycans in Decellularized Placenta Matrix as Critical Regulators for Cutaneous Wound Healing. Acta Biomater..

[55] Lobo S.E., Leonel L.C.P.C., Miranda C.M.F.C., Coelho T.M., Ferreira G.A.S., Mess A., Abrão M.S., Miglino M.A. (2016). The Placenta as an Organ and a Source of Stem Cells and Extracellular Matrix: A Review. Cells Tissues Organs.

[56] Ripellino J.A., Klinger M.M., Margolis R.U., Margolis R.K. (1985). The Hyaluronic Acid Binding Region as a Specific Probe for the Localization of Hyaluronic Acid in Tissue Sections. Application to Chick Embryo and Rat Brain. J. Histochem. Cytochem..

[57] Kleinman H.K., Sephel G.C., Tashiro K., Weeks B.S., Burrous B.A., Adler S.H., Yamada Y., Martin G.R. (1990). Laminin in Neuronal Development. Ann. N. Y. Acad. Sci..

[58] Schneider K.H., Aigner P., Holnthoner W., Monforte X., Nürnberger S., Rünzler D., Redl H., Teuschl A.H. (2016). Decellularized Human Placenta Chorion Matrix as a Favorable Source of Small-Diameter Vascular Grafts. Acta Biomater..

[59] Barros D., Amaral I.F., Pêgo A.P. (2020). Laminin-Inspired Cell-Instructive Microenvironments for Neural Stem Cells. Biomacromolecules.

[60] Nurcombe V. (1992). Laminin in Neural Development. Pharmacol. Ther..

[61] Yoshii S., Yamamuro T., Ito S., Hayashi M. (1987). In Vivo Guidance of Regenerating Nerve by Laminin-Coated Filaments. Exp. Neurol..

[62] Shan N., Zhang X., Xiao X., Zhang H., Tong C., Luo X., Chen Y., Liu X., Yin N., Deng Q. (2015). Laminin A4 (LAMA4) Expression Promotes Trophoblast Cell Invasion, Migration, and Angiogenesis, and Is Lowered in Preeclamptic Placentas. Placenta.

[63] Klaffky E., Williams R., Yao C.C., Ziober B., Kramer R., Sutherland A. (2001). Trophoblast-Specific Expression and Function of the Integrin Alpha 7 Subunit in the Peri-Implantation Mouse Embryo. Dev. Biol..

[64] Hall P.E., Lathia J.D., Caldwell M.A., Ffrench-Constant C. (2008). Laminin Enhances the Growth of Human Neural Stem Cells in Defined Culture Media. BMC Neurosci..

[65] Chen L.H., Xue J.F., Zheng Z.Y., Shuhaidi M., Thu H.E., Hussain Z. (2018). Hyaluronic Acid, an Efficient Biomacromolecule for Treatment of Inflammatory Skin and Joint Diseases: A Review of Recent Developments and Critical Appraisal of Preclinical and Clinical Investigations. Int. J. Biol. Macromol..

[66] Cooper C.A., Brown K.K., Meletis C.D., Zabriskie N. (2008). Inflammation and Hyaluronic Acid. Altern. Complement. Ther..

[67] Sunderland C.A., Bulmer J.N., Luscombe M., Redman C.W.G., Stirrat G.M. (1985). Immunohistological and Biochemical Evidence for a Role for Hyaluronic Acid in the Growth and Development of the Placenta. J. Reprod. Immunol..

[68] Peters A., Sherman L.S. (2020). Diverse Roles for Hyaluronan and Hyaluronan Receptors in the Developing and Adult Nervous System. Int. J. Mol. Sci..

[69] Seo T.B., Han I.S., Yoon J.H., Seol I.C., Kim Y.S., Jo H.K., An J.J., Hong K.E., Seo Y.B., Kim D.H. (2006). Growth-Promoting Activity of Hominis Placenta Extract on Regenerating Sciatic Nerve. Acta Pharmacol. Sin..

[70] Farhadi M., Gorji A., Mirsalehi M., Müller M., Poletaev A.B., Mahboudi F., Asadpour A., Ebrahimi M., Beiranvand M., Khaftari M.D. (2023). The Human Neuroprotective Placental Protein Composition Suppressing Tinnitus and Restoring Auditory Brainstem Response in a Rodent Model of Sodium Salicylate-Induced Ototoxicity. Heliyon.

[71] Park J.Y., Byeon J.H., Park S.W., Eun S.H., Chae K.Y., Eun B.L. (2013). Neuroprotective Effect of Human Placental Extract on Hypoxic-Ischemic Brain Injury in Neonatal Rats. Brain Dev..

[72] Mukherjee C., Saleem S., Das S., Biswas S.C., Bhattacharyya D. (2020). Human Placental Laminin: Role in Neuronal Differentiation, Cell Adhesion and Proliferation. J. Biosci..

[73] Nguyen B.P., Gil S.G., Carter W.G. (2000). Deposition of Laminin 5 by Keratinocytes Regulates Integrin Adhesion and Signaling. J. Biol. Chem..

[74] Hamada Y., Hirano E., Sugimoto K., Hanada K., Kaku T., Manda N., Tsuchida K. (2022). A Farewell to Phlebotomy-Use of Placenta-Derived Drugs Laennec and Porcine for Improving Hereditary Hemochromatosis without Phlebotomy: A Case Report. J. Med. Case Rep..

[75] Kong M., Park S.B. (2012). Effect of Human Placental Extract on Health Status in Elderly Koreans. Evid. Based Complement. Altern. Med..

[76] Roy A., Mantay M., Brannan C., Griffiths S. (2022). Placental Tissues as Biomaterials in Regenerative Medicine. Biomed. Res. Int..

[77] Kogure C., Tohda C. (2017). Human Placenta Extract Ameliorates Memory Dysfunction and Dendritic Atrophy in a 5XFAD Mouse Model of Alzheimer’s Disease. Tradit. Kampo Med..

[78] Jazayeri M.H., Barzaman K., Nedaeinia R., Aghaie T., Motallebnezhad M. (2020). Human Placental Extract Attenuates Neurological Symptoms in the Experimental Autoimmune Encephalomyelitis Model of Multiple Sclerosis-a Putative Approach in MS Disease?. Auto. Immun. Highlights.

[79] Shende P., Subedi M. (2017). Pathophysiology, Mechanisms and Applications of Mesenchymal Stem Cells for the Treatment of Spinal Cord Injury. Biomed. Pharmacother..

[80] Li Z., Zhao W., Liu W., Zhou Y., Jia J., Yang L. (2014). Transplantation of Placenta-Derived Mesenchymal Stem Cell-Induced Neural Stem Cells to Treat Spinal Cord Injury. Neural Regen. Res..

[81] Han S., Xiao Z., Li X., Zhao H., Wang B., Qiu Z., Li Z., Mei X., Xu B., Fan C. (2018). Human Placenta-Derived Mesenchymal Stem Cells Loaded on Linear Ordered Collagen Scaffold Improves Functional Recovery after Completely Transected Spinal Cord Injury in Canine. Sci. China Life Sci..

[82] Deng J., Li M., Meng F., Liu Z., Wang S., Zhang Y., Li M., Li Z., Zhang L., Tang P. (2021). 3D Spheroids of Human Placenta-Derived Mesenchymal Stem Cells Attenuate Spinal Cord Injury in Mice. Cell Death Dis..

[83] Wang A., Brown E.G., Lankford L., Keller B.A., Pivetti C.D., Sitkin N.A., Beattie M.S., Bresnahan J.C., Farmer D.L. (2015). Placental Mesenchymal Stromal Cells Rescue Ambulation in Ovine Myelomeningocele. Stem Cells Transl. Med..

[84] Kabagambe S., Keller B., Becker J., Goodman L., Pivetti C., Lankford L., Chung K., Lee C., Chen Y.J., Kumar P. (2018). Placental Mesenchymal Stromal Cells Seeded on Clinical Grade Extracellular Matrix Improve Ambulation in Ovine Myelomeningocele. J. Pediatr. Surg..

[85] Vanover M., Pivetti C., Lankford L., Kumar P., Galganski L., Kabagambe S., Keller B., Becker J., Chen Y.J., Chung K. (2019). High Density Placental Mesenchymal Stromal Cells Provide Neuronal Preservation and Improve Motor Function Following in Utero Treatment of Ovine Myelomeningocele. J. Pediatr. Surg..

[86] Galganski L.A., Kumar P., Vanover M.A., Pivetti C.D., Anderson J.E., Lankford L., Paxton Z.J., Chung K., Lee C., Hegazi M.S. (2020). In Utero Treatment of Myelomeningocele with Placental Mesenchymal Stromal Cells—Selection of an Optimal Cell Line in Preparation for Clinical Trials. J. Pediatr. Surg..

[87] Theodorou C.M., Stokes S.C., Jackson J.E., Pivetti C.D., Kumar P., Yamashiro K.J., Paxton Z.J., Reynaga L., Hyllen A.A., Wang A. (2022). Efficacy of Clinical-Grade Human Placental Mesenchymal Stromal Cells in Fetal Ovine Myelomeningocele Repair. J. Pediatr. Surg..

[88] Chen Y.J., Chung K., Pivetti C., Lankford L., Kabagambe S.K., Vanover M., Becker J., Lee C., Tsang J., Wang A. (2018). Fetal Surgical Repair with Placenta-Derived Mesenchymal Stromal Cell Engineered Patch in a Rodent Model of Myelomeningocele. J. Pediatr. Surg..

[89] Chung S., Rho S., Kim G., Kim S.-R., Baek K.-H., Kang M., Lew H. (2016). Human Umbilical Cord Blood Mononuclear Cells and Chorionic Plate-Derived Mesenchymal Stem Cells Promote Axon Survival in a Rat Model of Optic Nerve Crush Injury. Int. J. Mol. Med..

[90] Park M., Kim H.C., Kim O., Lew H. (2018). Human Placenta Mesenchymal Stem Cells Promote Axon Survival Following Optic Nerve Compression through Activation of NF-ΚB Pathway. J. Tissue Eng. Regen. Med..

[91] Kwon H., Park M., Nepali S., Lew H. (2020). Hypoxia-Preconditioned Placenta-Derived Mesenchymal Stem Cells Rescue Optic Nerve Axons Via Differential Roles of Vascular Endothelial Growth Factor in an Optic Nerve Compression Animal Model. Mol. Neurobiol..

[92] Park M., Kim H.-M., Shin H.-A., Lee S.-H., Hwang D.-Y., Lew H. (2021). Human Pluripotent Stem Cell-Derived Neural Progenitor Cells Promote Retinal Ganglion Cell Survival and Axon Recovery in an Optic Nerve Compression Animal Model. Int. J. Mol. Sci..

[93] Yun H.-M., Kim H.S., Park K.-R., Shin J.M., Kang A.R., il Lee K., Song S., Kim Y.-B., Han S.B., Chung H.-M. (2013). Placenta-Derived Mesenchymal Stem Cells Improve Memory Dysfunction in an Aβ1–42-Infused Mouse Model of Alzheimer’s Disease. Cell Death Dis..

[94] Kim K.Y., Suh Y.H., Chang K.A. (2020). Therapeutic Effects of Human Amniotic Epithelial Stem Cells in a Transgenic Mouse Model of Alzheimer’s Disease. Int. J. Mol. Sci..

[95] Jiao H., Shi K., Zhang W., Yang L., Yang L., Guan F., Yang B. (2016). Therapeutic Potential of Human Amniotic Membrane-Derived Mesenchymal Stem Cells in APP Transgenic Mice. Oncol. Lett..

[96] Kim K.S., Kim H.S., Park J.M., Kim H.W., Park M.-K., Lee H.S., Lim D.S., Lee T.H., Chopp M., Moon J. (2013). Long-Term Immunomodulatory Effect of Amniotic Stem Cells in an Alzheimer’s Disease Model. Neurobiol. Aging.

[97] Sun H., Hou Z., Yang H., Meng M., Li P., Zou Q., Yang L., Chen Y., Chai H., Zhong H. (2014). Multiple Systemic Transplantations of Human Amniotic Mesenchymal Stem Cells Exert Therapeutic Effects in an ALS Mouse Model. Cell Tissue Res..

[98] Kho A.R., Kim O.J., Jeong J.H., Yu J.M., Kim H.S., Choi B.Y., Suh S.W., Chung T.N. (2018). Administration of Placenta-Derived Mesenchymal Stem Cells Counteracts a Delayed Anergic State Following a Transient Induction of Endogenous Neurogenesis Activity after Global Cerebral Ischemia. Brain Res..

[99] González P.L., Carvajal C., Cuenca J., Alcayaga-Miranda F., Figueroa F.E., Bartolucci J., Salazar-Aravena L., Khoury M. (2015). Chorion Mesenchymal Stem Cells Show Superior Differentiation, Immunosuppressive, and Angiogenic Potentials in Comparison With Haploidentical Maternal Placental Cells. Stem Cells Transl. Med..

[100] Kmiecik G., Niklińska W., Kuć P., Pancewicz-Wojtkiewicz J., Fil D., Karwowska A., Karczewski J., Mackiewicz Z. (2013). Fetal Membranes as a Source of Stem Cells. Adv. Med. Sci..

